# 2-Hy­droxy­ethanaminium 2-methyl-5-nitro­benzene­sulfonate

**DOI:** 10.1107/S160053681203382X

**Published:** 2012-08-31

**Authors:** Peng Fei Hu, Ying Zheng, Wen Xi Wang

**Affiliations:** a College of Chemical Engineering and Material Sciences, Zhejiang University of Technology, Hangzhou 310014, People’s Republic of China; b Department of Pharmacy, 117th Hospital of PLA, Hangzhou 310013, People’s Republic of China; c College of Pharmaceutical Sciences, Zhejiang University of Technology, Hangzhou 310014, People’s Republic of China

## Abstract

In the crystal structure of the title salt, C_2_H_8_NO^+^·C_7_H_6_NO_5_S^−^, the cations and anions are linked together by N—H⋯O and O—H⋯O hydrogen bonds, forming layers parallel to (100). The plane of nitro group is skew with respect to the plane of benzene ring, making a dihedral angle of 17.5 (2)°.

## Related literature

For the structures of pyridinium derivative, nickel, magnesium and potassium salts of 2-methyl-5-nitrobenzenesulfonate, see, respectively: Gu *et al.* (2007 ▶); Xie *et al.* (2007 ▶); Xie, Lui & Yuan (2006 ▶); Xie, Yang *et al.* (2006 ▶).
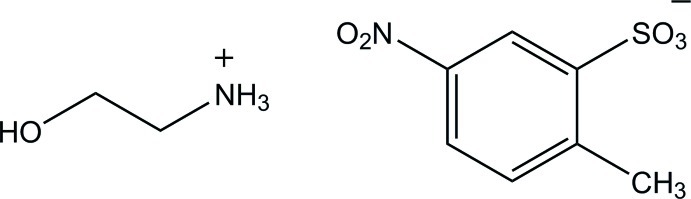


## Experimental

### 

#### Crystal data

C_2_H_8_NO^+^·C_7_H_6_NO_5_S^−^
*M* *_r_* = 278.29
Monoclinic, 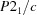

*a* = 14.8130 (5) Å
*b* = 9.5617 (4) Å
*c* = 8.6697 (3) Å
β = 103.071 (1)°
*V* = 1196.14 (8) Å^3^
*Z* = 4
Mo *K*α radiation
μ = 0.29 mm^−1^
*T* = 293 K
0.32 × 0.30 × 0.28 mm

#### Data collection

Bruker SMART APEX CCD diffractometer
Absorption correction: multi-scan (*SADABS*; Bruker, 2002 ▶) *T*_min_ = 0.873, *T*_max_ = 0.910
11263 measured reflections
2739 independent reflections
2620 reflections with *I* > 2σ(*I*)
*R*_int_ = 0.032

#### Refinement

*R*[*F* ^2^ > 2σ(*F* ^2^)] = 0.036
*wR*(*F* ^2^) = 0.095
*S* = 1.11
2739 reflections
181 parameters
1 restraint
H atoms treated by a mixture of independent and constrained refinement
Δρ_max_ = 0.28 e Å^−3^
Δρ_min_ = −0.39 e Å^−3^

### 

Data collection: *SMART* (Bruker, 2007 ▶); cell refinement: *SAINT* (Bruker, 2007 ▶); data reduction: *SAINT*; program(s) used to solve structure: *SHELXS97* (Sheldrick, 2008 ▶); program(s) used to refine structure: *SHELXL97* (Sheldrick, 2008 ▶); molecular graphics: *SHELXTL* (Sheldrick, 2008 ▶); software used to prepare material for publication: *SHELXL97*.

## Supplementary Material

Crystal structure: contains datablock(s) global, I. DOI: 10.1107/S160053681203382X/is5129sup1.cif

Structure factors: contains datablock(s) I. DOI: 10.1107/S160053681203382X/is5129Isup2.hkl

Supplementary material file. DOI: 10.1107/S160053681203382X/is5129Isup3.cml

Additional supplementary materials:  crystallographic information; 3D view; checkCIF report

## Figures and Tables

**Table 1:** Hydrogen-bond geometry (Å, °)

*D*—H⋯*A*	*D*—H	H⋯*A*	*D*⋯*A*	*D*—H⋯*A*
N2—H2*A*⋯O5^i^	0.89 (2)	2.04 (2)	2.872 (2)	156 (2)
N2—H2*B*⋯O5	0.85 (2)	2.10 (2)	2.938 (2)	166 (2)
N2—H2*C*⋯O3^ii^	0.92 (2)	2.00 (2)	2.914 (1)	172 (2)
O6—H6′⋯O4^iii^	0.81 (1)	1.97 (1)	2.760 (1)	165 (1)

Symmetry codes: (i) 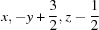; (ii) 
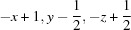
; (iii) 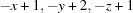.

**Fig. 1.**
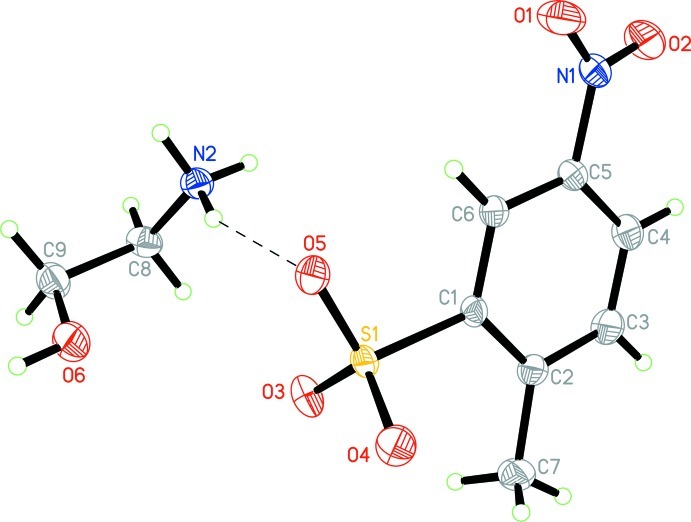
The asymmetric unit of the title compound with labeling and displacement ellipsoids drawn at the 40% probability level. One N—H···O hydrogen bond is illustrated as a dashed line.

**Fig. 2.**
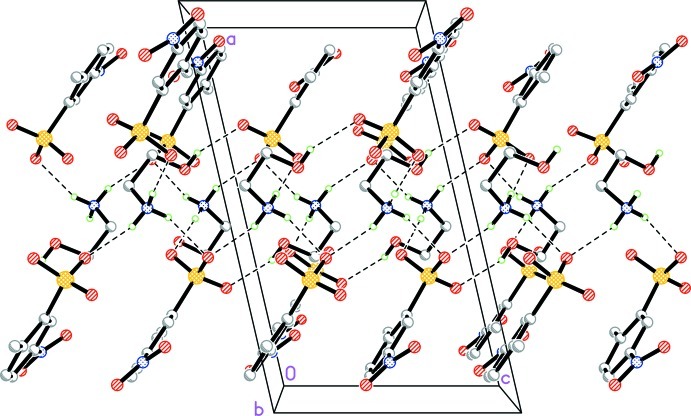
The hydrogen bonding layer of the title compound viewed down along the b axis. Hydrogen bonds are drawn as dashed lines. The H atoms not involved in the hydrogen bonds have been omitted for clarity.

## supplementary crystallographic information

### Comment

A few crystal structures containing 2-methyl-5-nitrobenzenesulfonate have been reported previously (Gu *et al.*, 2007; Xie *et al.*, 2007; Xie, Lui & Yuan, 2006; Xie, Yang *et al.*, 2006).

In the asymmetric unit of the title compound, the 2-ethanolamine molecule is protonated and the 2-methyl-5-nitrobenzenesulfonic acid molecule loses its acid H atom, then they are linked by an N2—H2B···O5 hydrogen bond (Fig. 1 & Table 1). The plane of nitro group is skew with the plane of benzene ring in a dihedral angle of 17.5 (2)°. The C1—C2 bond [1.4054 (16) Å] is the longest one among the other aromatic C—C bond, this is consistent with the situations observed in the previous cases (1.405 Å, Gu *et al.*, 2007; 1.404 Å, Xie, Lui & Yuan, 2006; 1.407 Å, Xie *et al.*, 2007; 1.408 Å, Xie, Yang *et al.*, 2006).

A few crystal structures containing 2-methyl-5-nitrobenzenesulfonate have been reported previously, they are pyridinium derivative (Gu *et al.*, 2007), nickel (Xie, Yang *et al.*, 2006), magnesium (Xie *et al.*, 2007), and potassium salts (Xie, Lui & Yuan, 2006). In the potassium salt, all of the oxygen atoms of sulfonate and one oxygen atom of the nitro group is coordinated with potassium atom directly. However, there exists no covalent bond between the counter ion pair in the title compound, which is similar with the other three previous cases. In all of these cases, one of C-C bond of benzene rings are slightly abormal.

In the crystal, the 2-hydroxyethanaminium cations and the MNB anions are linked by N—H···O and O—H···O hydrogen bonds (Table 1) to form thick layers (Fig. 2) parallel to the (100) plane. The nearest separation between the centroid of MNB benzene rings is of 4.483 (3) Å, suggesting no π–π interaction. This situation is similar to that observed in the case containing a large sized organic cation as counter ion for MNB anion (Gu *et al.*, 2007), but is different from those observed in other cases containing metal cations as counter ion for MNB anion (Xie *et al.*, 2007; Xie, Lui & Yuan, 2006; Xie, Yang *et al.*, 2006).

### Experimental

2-Methyl-5-nitrobenzenesulfonic acid (12.1 g) and 2-ethanolamine (5.0 g) were mixed and dissolved in sufficient water (25 ml) by heating to 373 K, at which point a clear solution resulted. The solution was then cooled slowly to room temperature. Crystals of the title compound (9.2 g) were formed upon the evaporation of water, then collected and washed with ethanol.

### Refinement

All H atoms of hydroxyl and ammonium groups were located in a difference Fourier map. The H atoms of ammonium group were refined freely, but the H atom of hydroxyl group was refined with a distance restraint O—H = 0.82 (1) Å, and with *U*_iso_(H) = 1.5*U*_eq_(O). Other H atoms were placed in calculated positions (C—H = 0.93–0.97 Å) and allowed to ride on their parent atoms, with *U*_iso_(H) = 1.2*U*_eq_(C).

### Crystal data

**Table:**

C_2_H_8_NO^+^·C_7_H_6_NO_5_S^−^	*F*(000) = 584
*M**_r_* = 278.29	*D*_x_ = 1.545 Mg m^−^^3^
Monoclinic, *P*2_1_/*c*	Melting point < 424 K
Hall symbol: -P 2ybc	Mo *K*α radiation, λ = 0.71073 Å
*a* = 14.8130 (5) Å	Cell parameters from 1766 reflections
*b* = 9.5617 (4) Å	θ = 1.6–17.6°
*c* = 8.6697 (3) Å	µ = 0.29 mm^−^^1^
β = 103.071 (1)°	*T* = 293 K
*V* = 1196.14 (8) Å^3^	Prism, colorless
*Z* = 4	0.32 × 0.30 × 0.28 mm

### Data collection

**Table:**

Bruker SMART APEX CCD diffractometer	2739 independent reflections
Radiation source: sealed tube	2620 reflections with *I* > 2σ(*I*)
Graphite monochromator	*R*_int_ = 0.032
φ and ω scans	θ_max_ = 27.5°, θ_min_ = 3.2°
Absorption correction: multi-scan (*SADABS*; Bruker, 2002)	*h* = −19→19
*T*_min_ = 0.873, *T*_max_ = 0.910	*k* = −12→12
11263 measured reflections	*l* = −11→11

### Refinement

**Table:**

Refinement on *F*^2^	Secondary atom site location: difference Fourier map
Least-squares matrix: full	Hydrogen site location: inferred from neighbouring sites
*R*[*F*^2^ > 2σ(*F*^2^)] = 0.036	H atoms treated by a mixture of independent and constrained refinement
*wR*(*F*^2^) = 0.095	*w* = 1/[σ^2^(*F*_o_^2^) + (0.0532*P*)^2^ + 0.342*P*] where *P* = (*F*_o_^2^ + 2*F*_c_^2^)/3
*S* = 1.11	(Δ/σ)_max_ = 0.001
2739 reflections	Δρ_max_ = 0.28 e Å^−^^3^
181 parameters	Δρ_min_ = −0.39 e Å^−^^3^
1 restraint	Extinction correction: *SHELXL97* (Sheldrick, 2008), Fc^*^=kFc[1+0.001xFc^2^λ^3^/sin(2θ)]^-1/4^
Primary atom site location: structure-invariant direct methods	Extinction coefficient: 0.095 (5)

### Special details

**Table:**

Geometry. All e.s.d.'s (except the e.s.d. in the dihedral angle between two l.s. planes) are estimated using the full covariance matrix. The cell e.s.d.'s are taken into account individually in the estimation of e.s.d.'s in distances, angles and torsion angles; correlations between e.s.d.'s in cell parameters are only used when they are defined by crystal symmetry. An approximate (isotropic) treatment of cell e.s.d.'s is used for estimating e.s.d.'s involving l.s. planes.
Refinement. Refinement of *F*^2^ against ALL reflections. The weighted *R*-factor *wR* and goodness of fit *S* are based on *F*^2^, conventional *R*-factors *R* are based on *F*, with *F* set to zero for negative *F*^2^. The threshold expression of *F*^2^ > σ(*F*^2^) is used only for calculating *R*-factors(gt) *etc*. and is not relevant to the choice of reflections for refinement. *R*-factors based on *F*^2^ are statistically about twice as large as those based on *F*, and *R*- factors based on ALL data will be even larger.

### Fractional atomic coordinates and isotropic or equivalent isotropic displacement parameters (Å^2^)

**Table:**

	*x*	*y*	*z*	*U*_iso_*/*U*_eq_	
S1	0.320970 (19)	0.93470 (3)	0.27057 (3)	0.02240 (13)	
O1	0.16252 (9)	0.44692 (11)	0.12844 (15)	0.0467 (3)	
O2	0.07025 (8)	0.46497 (13)	−0.10098 (15)	0.0484 (3)	
O3	0.37472 (7)	1.00898 (11)	0.17553 (11)	0.0346 (2)	
O4	0.28710 (7)	1.02587 (12)	0.37875 (11)	0.0359 (3)	
O5	0.37141 (7)	0.81368 (10)	0.35012 (11)	0.0328 (2)	
N1	0.12202 (8)	0.51582 (12)	0.01568 (14)	0.0321 (3)	
C1	0.22210 (8)	0.86643 (13)	0.13486 (13)	0.0217 (2)	
C2	0.15933 (9)	0.95606 (13)	0.03584 (15)	0.0265 (3)	
C3	0.08519 (9)	0.89425 (16)	−0.07087 (16)	0.0339 (3)	
H3	0.0427	0.9514	−0.1376	0.041*	
C4	0.07288 (9)	0.75096 (16)	−0.08063 (16)	0.0328 (3)	
H4	0.0236	0.7118	−0.1537	0.039*	
C5	0.13542 (8)	0.66756 (13)	0.02065 (15)	0.0262 (3)	
C6	0.21014 (8)	0.72276 (13)	0.12945 (14)	0.0243 (3)	
H6	0.2513	0.6646	0.1973	0.029*	
C7	0.16737 (11)	1.11277 (15)	0.04080 (19)	0.0385 (3)	
H7A	0.1641	1.1457	0.1440	0.046*	
H7B	0.1176	1.1527	−0.0372	0.046*	
H7C	0.2256	1.1400	0.0188	0.046*	
O6	0.59657 (8)	0.94383 (12)	0.32575 (13)	0.0407 (3)	
N2	0.49673 (8)	0.71679 (12)	0.15377 (14)	0.0282 (2)	
C8	0.55294 (10)	0.81663 (14)	0.08550 (15)	0.0304 (3)	
H8A	0.5137	0.8929	0.0360	0.037*	
H8B	0.5771	0.7698	0.0040	0.037*	
C9	0.63225 (10)	0.87505 (16)	0.20792 (17)	0.0351 (3)	
H9A	0.6734	0.8001	0.2552	0.042*	
H9B	0.6673	0.9405	0.1589	0.042*	
H6'	0.6382 (11)	0.954 (2)	0.4039 (17)	0.048 (5)*	
H2A	0.4547 (16)	0.683 (2)	0.072 (3)	0.058 (6)*	
H2B	0.4688 (14)	0.753 (2)	0.220 (2)	0.046 (5)*	
H2C	0.5324 (13)	0.646 (2)	0.208 (2)	0.044 (5)*	

### Atomic displacement parameters (Å^2^)

**Table:**

	*U*^11^	*U*^22^	*U*^33^	*U*^12^	*U*^13^	*U*^23^
S1	0.01922 (18)	0.02454 (19)	0.02070 (18)	−0.00232 (10)	−0.00124 (12)	−0.00248 (10)
O1	0.0523 (7)	0.0270 (5)	0.0529 (7)	−0.0034 (5)	−0.0043 (6)	0.0035 (5)
O2	0.0415 (6)	0.0386 (6)	0.0560 (7)	−0.0100 (5)	−0.0078 (5)	−0.0182 (5)
O3	0.0285 (5)	0.0414 (6)	0.0327 (5)	−0.0116 (4)	0.0039 (4)	0.0007 (4)
O4	0.0347 (5)	0.0421 (6)	0.0287 (5)	0.0010 (4)	0.0021 (4)	−0.0130 (4)
O5	0.0274 (5)	0.0326 (5)	0.0316 (5)	0.0020 (4)	−0.0079 (4)	0.0026 (4)
N1	0.0255 (5)	0.0279 (6)	0.0408 (6)	−0.0046 (4)	0.0030 (5)	−0.0070 (5)
C1	0.0180 (5)	0.0247 (6)	0.0207 (5)	−0.0007 (4)	0.0005 (4)	−0.0020 (4)
C2	0.0238 (6)	0.0258 (6)	0.0273 (6)	0.0019 (5)	0.0003 (5)	0.0003 (5)
C3	0.0266 (6)	0.0339 (7)	0.0340 (7)	0.0046 (5)	−0.0082 (5)	0.0023 (5)
C4	0.0245 (6)	0.0359 (7)	0.0319 (6)	−0.0018 (5)	−0.0065 (5)	−0.0061 (5)
C5	0.0224 (5)	0.0250 (6)	0.0292 (6)	−0.0026 (5)	0.0019 (5)	−0.0045 (5)
C6	0.0208 (5)	0.0249 (6)	0.0248 (5)	0.0001 (4)	0.0001 (4)	−0.0008 (4)
C7	0.0369 (7)	0.0250 (7)	0.0477 (8)	0.0030 (6)	−0.0031 (6)	0.0036 (6)
O6	0.0391 (6)	0.0446 (6)	0.0330 (6)	0.0013 (5)	−0.0030 (5)	−0.0150 (4)
N2	0.0305 (6)	0.0258 (5)	0.0254 (5)	0.0002 (4)	0.0003 (5)	−0.0021 (4)
C8	0.0391 (7)	0.0272 (6)	0.0230 (6)	−0.0027 (5)	0.0030 (5)	−0.0006 (5)
C9	0.0321 (6)	0.0364 (7)	0.0354 (7)	−0.0045 (6)	0.0045 (6)	−0.0058 (6)

### Geometric parameters (Å, º)

**Table:**

S1—O4	1.4506 (10)	C6—H6	0.9300
S1—O3	1.4540 (10)	C7—H7A	0.9600
S1—O5	1.4628 (10)	C7—H7B	0.9600
S1—C1	1.7821 (11)	C7—H7C	0.9600
O1—N1	1.2183 (16)	O6—C9	1.4141 (17)
O2—N1	1.2234 (15)	O6—H6'	0.813 (9)
N1—C5	1.4638 (16)	N2—C8	1.4757 (17)
C1—C6	1.3846 (17)	N2—H2A	0.89 (2)
C1—C2	1.4054 (16)	N2—H2B	0.85 (2)
C2—C3	1.3971 (18)	N2—H2C	0.92 (2)
C2—C7	1.5030 (18)	C8—C9	1.5016 (18)
C3—C4	1.382 (2)	C8—H8A	0.9700
C3—H3	0.9300	C8—H8B	0.9700
C4—C5	1.3774 (19)	C9—H9A	0.9700
C4—H4	0.9300	C9—H9B	0.9700
C5—C6	1.3860 (16)		
			
O4—S1—O3	112.75 (7)	C5—C6—H6	120.7
O4—S1—O5	112.65 (6)	C2—C7—H7A	109.5
O3—S1—O5	111.52 (6)	C2—C7—H7B	109.5
O4—S1—C1	107.11 (6)	H7A—C7—H7B	109.5
O3—S1—C1	106.16 (5)	C2—C7—H7C	109.5
O5—S1—C1	106.10 (6)	H7A—C7—H7C	109.5
O1—N1—O2	123.46 (13)	H7B—C7—H7C	109.5
O1—N1—C5	118.26 (11)	C9—O6—H6'	108.8 (15)
O2—N1—C5	118.28 (12)	C8—N2—H2A	105.8 (14)
C6—C1—C2	121.49 (11)	C8—N2—H2B	114.3 (14)
C6—C1—S1	117.75 (9)	H2A—N2—H2B	108.7 (19)
C2—C1—S1	120.75 (9)	C8—N2—H2C	111.7 (12)
C3—C2—C1	117.32 (12)	H2A—N2—H2C	111.0 (19)
C3—C2—C7	119.08 (12)	H2B—N2—H2C	105.5 (18)
C1—C2—C7	123.59 (11)	N2—C8—C9	112.29 (11)
C4—C3—C2	122.21 (12)	N2—C8—H8A	109.1
C4—C3—H3	118.9	C9—C8—H8A	109.1
C2—C3—H3	118.9	N2—C8—H8B	109.1
C5—C4—C3	118.32 (12)	C9—C8—H8B	109.1
C5—C4—H4	120.8	H8A—C8—H8B	107.9
C3—C4—H4	120.8	O6—C9—C8	108.85 (12)
C4—C5—C6	122.13 (12)	O6—C9—H9A	109.9
C4—C5—N1	119.20 (12)	C8—C9—H9A	109.9
C6—C5—N1	118.66 (11)	O6—C9—H9B	109.9
C1—C6—C5	118.51 (11)	C8—C9—H9B	109.9
C1—C6—H6	120.7	H9A—C9—H9B	108.3

### Hydrogen-bond geometry (Å, º)

**Table:**

*D*—H···*A*	*D*—H	H···*A*	*D*···*A*	*D*—H···*A*
N2—H2*A*···O5^i^	0.89 (2)	2.04 (2)	2.872 (2)	156 (2)
N2—H2*B*···O5	0.85 (2)	2.10 (2)	2.938 (2)	166 (2)
N2—H2*C*···O3^ii^	0.92 (2)	2.00 (2)	2.914 (1)	172 (2)
O6—H6′···O4^iii^	0.81 (1)	1.97 (1)	2.760 (1)	165 (1)

Symmetry codes: (i) *x*, −*y*+3/2, *z*−1/2; (ii) −*x*+1, *y*−1/2, −*z*+1/2; (iii) −*x*+1, −*y*+2, −*z*+1.
